# Antinociceptive Effects of Free and β-Cyclodextrin-Associated α-Phellandrene in CFA-Induced Inflammatory Pain

**DOI:** 10.3390/biomedicines14091932

**Published:** 2026-08-28

**Authors:** Ana Rita de Sousa França, Wilmara de Carvalho Santos, Matheus Rocha de Seixas Nogueira, Elza Mayara Antunes de Macedo Andrade, Antonio Carlos dos Reis Filho, Eduardo Lima Feitosa, Sidney Gonçalo de Lima, Francisco das Chagas Alves Lima, Francisco Ivan da Silva, Damião Pergentino de Sousa, Antonia Laíres da Silva Santos, Francisco de Assis Oliveira, Fernanda Regina de Castro Almeida

**Affiliations:** 1 Medicinal Plants Research Center, Federal University of Piauí, Avenida Nossa Senhora de Fátima s/n, Teresina 64049-550, PI, Brazil; francaanarita@gmail.com (A.R.d.S.F.); matheusrsn@gmail.com (M.R.d.S.N.); elza.mayara@hotmail.com (E.M.A.d.M.A.); carlosfilho_089@hotmail.com (A.C.d.R.F.); antonia.laires@ufpi.edu.br (A.L.d.S.S.); 2 Federal Institute of Education, Science and Technology of Maranhão (IFMA), Timon Campus, Timon 65635-468, MA, Brazil; wilmara.santos@ifma.edu.br; 3 Organic Geochemistry Laboratory, Federal University of Piauí, Avenida Nossa Senhora de Fátima s/n, Teresina 64049-550, PI, Brazil; edu-if@hotmail.com (E.L.F.); sidney@ufpi.edu.br (S.G.d.L.); 4 Department of Chemistry, State University of Piauí, Rua João Cabral, 2231, Pirajá, Teresina 64002-150, PI, Brazil; fdcalima@ccn.uespi.br; 5 Federal Institute of Education, Science and Technology of Piauí (IFPI), Paulistana Campus, Paulistana 64750-000, PI, Brazil; ivanchemistry007@ifpi.edu.br; 6 Department of Pharmaceutical Sciences, Federal University of Paraíba, University City, s/n, João Pessoa 58051-900, PB, Brazil; damiao_desousa@yahoo.com.br

**Keywords:** α-phellandrene, monoterpene, cyclodextrin-based preparation, β-cyclodextrin, antinociceptive, inflammatory pain, Complete Freund’s adjuvant

## Abstract

**Background/Objectives:** α-Phellandrene (α-PHEL) is a monoterpene found in essential oils and is known for its antinociceptive and anti-inflammatory properties. This study investigated the antinociceptive effects of free α-PHEL and β-cyclodextrin-associated α-PHEL (α-PHEL/β-CD) in a Complete Freund’s adjuvant (CFA)-induced model of chronic inflammatory pain. **Methods:** Mechanical allodynia and hyperalgesia were evaluated in female Wistar rats using the von Frey and Randall–Selitto tests, respectively. Locomotor activity and motor performance were evaluated in male Swiss mice. Animals received α-PHEL (6.25–100 mg/kg) and α-PHEL/β-CD (3.12–12.5 mg/kg), as well as vehicle (2% Tween 80/0.9% saline) and dexamethasone (0.5 mg/kg, p.o.), with evaluations conducted in the von Frey test at intervals of 1 to 24 h. The antihyperalgesic effect was evaluated using α-PHEL, α-PHEL/β-CD, vehicle, and diclofenac (5 mg/kg, p.o.), with assessments in the Randall–Selitto test from 1 to 6 h. Over 10 days, the animals were treated and evaluated daily to determine the effect during repeated treatment. The involvement of the opioid pathway was assessed using naloxone, whereas the serotonergic pathway was investigated using PCPA and ketanserin in the von Frey test. Motor coordination was tested in the rotarod, and exploratory behavior was assessed in the open field. **Results:** α-PHEL reduced CFA-induced hyperalgesia and allodynia in both acute and chronic phases. The α-PHEL/β-CD preparation produced significant antinociceptive effects at all tested doses during repeated administration. Naloxone and 5-HT_2_A receptor blockade reduced the antinociceptive effect of α-PHEL, while α-PHEL and the α-PHEL/β-CD preparation did not alter locomotor activity or motor coordination. **Conclusions:** The α-PHEL/β-CD preparation exhibits antinociceptive activity in inflammatory pain without affecting locomotor activity or motor coordination. The findings with free α-PHEL suggest the possible contribution of opioid and serotonergic pathways to its antinociceptive effect.

## 1. Introduction

Inflammatory pain is one of the most prevalent clinical conditions and represents a major therapeutic challenge, particularly when it progresses to chronic stages and significantly impairs patients’ quality of life [1,2]. Persistent inflammatory pain is commonly associated with tissue injury and the subsequent release of inflammatory mediators that promote peripheral and central sensitization of nociceptive pathways [3,4]. This sensitization process reduces the activation threshold of nociceptive neurons, contributing to the development of hyperalgesia and allodynia, two hallmark features of chronic inflammatory pain states [5,6,7].

Current pharmacological approaches for inflammatory pain management mainly include nonsteroidal anti-inflammatory drugs (NSAIDs) and opioids [8,9]. Although these drugs are effective in reducing pain and inflammation, their prolonged use is frequently associated with important adverse effects. NSAIDs may induce gastrointestinal, cardiovascular, and renal complications [10,11], whereas opioids are strongly associated with tolerance, dependence, constipation, sedation, and other central nervous system-related effects [9,12]. These limitations highlight the need for the development of safer and more effective therapeutic alternatives for chronic inflammatory pain.

Natural products, particularly essential oils and their bioactive constituents, have attracted increasing attention as potential sources of novel analgesic and anti-inflammatory agents [13]. Among these compounds, monoterpenes have demonstrated relevant pharmacological activities, including antinociceptive, anti-inflammatory, and antihyperalgesic effects in several experimental models [14,15,16,17].

α-Phellandrene (α-PHEL), a cyclic monoterpene present in the essential oils of several medicinal plants, including *Rosmarinus officinalis* L. (syn. *Salvia rosmarinus* Spenn.), *Callistemon citrinus*, *Curcuma longa* L., and *Unxia camphorata* L. f. [18,19,20,21], has emerged as a promising compound with anti-inflammatory and antinociceptive properties. Previous studies have shown that α-PHEL reduces inflammatory cell migration, inhibits mast cell degranulation, and attenuates nociceptive responses in acute pain models [22,23]. In addition, its antinociceptive effects appear to involve multiple mechanisms, including modulation of opioid, nitrergic, cholinergic, and adrenergic pathways, without inducing motor impairment or apparent toxicity [23]. Despite these promising pharmacological properties, the therapeutic application of α-PHEL may be limited by the low aqueous solubility and bioavailability commonly associated with monoterpenes.

Cyclodextrins (CDs) are cyclic oligosaccharides widely used to improve the physicochemical and pharmacological properties of lipophilic compounds through inclusion complex formation [24]. Among them, β-cyclodextrin (β-CD) has been widely investigated due to its ability to enhance the solubility, stability, bioavailability, and biological activity of several natural products [25]. Previous studies have demonstrated that complexation with β-CD potentiates the antinociceptive effects of terpenes and other bioactive compounds in different pain models [25].

Therefore, considering the pharmacological potential of α-PHEL and the advantages provided by β-CD complexation, the present study extends previous findings by evaluating free α-PHEL and the α-PHEL/β-CD system following acute and repeated administration in a CFA-induced model of persistent inflammatory pain. The possible contribution of opioid and serotonergic pathways to the antinociceptive effect of free α-PHEL was also investigated.

## 2. Materials and Methods

The following substances were used. α-Phellandrene (α-PHEL) was purchased from Sigma-Aldrich (St. Louis, MO, USA) as a mixture enriched with 80% (-)-α-phellandrene, according to the manufacturer’s specifications. β-Cyclodextrin (β-CD), carrageenan type I, morphine, naloxone, *p*-chlorophenylalanine (PCPA), ketanserin, and analytical-grade ethanol were obtained from Sigma-Aldrich (St. Louis, MO, USA). Complete Freund’s adjuvant (CFA) was purchased from Difco (Detroit, MI, USA). Indomethacin and diclofenac sodium were obtained from Galen (Campinas, SP, Brazil). Diazepam was purchased from Cristália (Itapira, SP, Brazil), and methanol was obtained from Cryslab (Teresina, PI, Brazil).

For the pharmacological assays, α-PHEL was suspended in 2% Tween 80 in 0.9% saline (10 mL/kg), which served as its control. β-CD (12.5 mg/kg, p.o.) was used as the control for the α-PHEL/β-CD preparation. All doses were expressed in mg/kg according to body weight.

### 2.1. Preparation of the α-Phellandrene/β-Cyclodextrin System

The α-phellandrene/β-cyclodextrin (α-PHEL/β-CD) system was prepared according to the method described by Fernandes et al. [26], with modifications. Initially, α-PHEL (1 g) was dissolved in 20 mL of analytical-grade ethanol, while β-cyclodextrin (9 g) was dissolved in 100 mL of distilled water at 45 °C, corresponding to an approximate 1:1 molar ratio (α-PHEL:β-CD). Subsequently, the α-PHEL solution was slowly added to the β-CD solution under constant magnetic stirring at 600 rpm for 90 min at room temperature (25 ± 2 °C) and protected from light. The resulting mixture was then dried using a spray dryer (BÜCHI B-290, Flawil, Switzerland) under the following operating conditions: inlet temperature of 120 °C, outlet temperature of 75 ± 5 °C, feed flow rate of 5 mL/min, aspiration rate of 85%, and airflow rate of 600 L/h, to obtain the spray-dried α-PHEL/β-CD preparation.

### 2.2. Complexation Efficiency

The α-PHEL content in the spray-dried α-PHEL/β-CD preparation was determined by UV–Vis spectrophotometry. Initially, a 30 μg/mL solution of α-PHEL in methanol was scanned from 200 to 400 nm at 1 nm intervals using a Shimadzu UV-1800 spectrophotometer (Shimadzu Corporation, Kyoto, Japan) to determine its absorption characteristics. A calibration curve was subsequently constructed using α-PHEL solutions in methanol at concentrations of 3.12, 6.25, 12.5, 25, and 50 μg/mL. Absorbance was measured at 264 nm using a Bioespectro SP-220 UV–Vis spectrophotometer (BioSpectro, Curitiba, PR, Brazil).

Three independent analyses were performed within the linear concentration range. α-PHEL was exhaustively extracted from the β-CD matrix three times using methanol under mild heating. The extracts were filtered through a 0.22 μm cellulose acetate membrane and quantified spectrophotometrically at 264 nm.

Encapsulation efficiency (EE) and loading efficiency (LE) were calculated according to Equations (1) and (2), respectively:

EE (%) = (Amount of α-PHEL recovered from the β-CD matrix/Initial amount of α-PHEL) × 100(1)

LE (%) = (Amount of α-PHEL recovered from the β-CD matrix/Total mass of the spray-dried preparation) × 100(2)

Encapsulation efficiency was calculated as the percentage of α-PHEL recovered from the β-CD matrix relative to the initial amount used during preparation. Loading efficiency was calculated as the amount of α-PHEL recovered from the final spray-dried preparation relative to its total mass.

### 2.3. Computational Modeling

Molecular modeling analyses were performed to investigate the possible stability and orientation of the theoretical host–guest system formed between β-cyclodextrin (β-CD) and α-phellandrene (α-PHEL) at a 1:1 molar ratio. Four different orientations of α-PHEL within the β-CD cavity were evaluated, corresponding to distinct host–guest conformations generated according to the possible insertion modes of the monoterpene into the cyclodextrin cavity.

Geometry optimization and vibrational frequency calculations were carried out to obtain thermodynamic parameters, including Gibbs free energy (ΔG), in order to compare the theoretical stability of the evaluated host–guest configurations.

All theoretical calculations were performed in vacuum using the Gaussian 09W Revision D.01 computational package with the semiempirical PM6 method [27], due to its suitability for host–guest interaction studies and low computational cost. Neutral systems with singlet multiplicity were considered throughout the calculations. The optimized molecular structures were visualized using the GaussView 5.0 software interface.

### 2.4. Infrared Spectroscopy (IR)

To analyze the infrared spectra of α-PHEL, β-CD, the physical mixture, and the spray-dried α-PHEL/β-CD preparation, a Vertex 70 spectrometer (Bruker Optics GmbH & Co. KG, Ettlingen, Germany) equipped with an attenuated total reflectance (ATR) accessory was employed. Spectra were recorded in the mid-infrared region (4000 to 600 cm^−1^). Approximately 20 μL of α-PHEL and 5 mg each of β-CD, the physical mixture, and the α-PHEL/β-CD preparation were placed on the germanium (Ge) crystal surface of the ATR accessory, with slight pressure applied to ensure adequate contact between the samples and the crystal. Spectra were acquired from 64 scans at a resolution of 4 cm^−1^. The infrared spectra were smoothed, and baseline correction was performed using standard FTIR spectral preprocessing procedures [28].

### 2.5. Animals

The study utilized female Wistar rats (170–230 g) and male Swiss mice (25–30 g), procured from the Medicinal Plants Research Center at the Federal University of Piauí. The animals were approximately 2 months old at the time of the experiments. Animals were allocated to the experimental groups before the start of the experiments; no formal randomization sequence was used. The investigators were aware of the treatment allocation during behavioral assessments and data analysis. The animals were housed under controlled laboratory conditions (22 ± 1 °C) with a 12 h light–dark cycle and had access to food and water ad libitum. Prior to testing, the animals were acclimatized in the laboratory for at least one hour. The experimental unit was one individual animal. Sample sizes were determined based on previous studies using the same experimental model and treatment protocol. No formal a priori power calculation was performed. No animals or experimental data were excluded from the statistical analyses. All experimental protocols were approved by the Institutional Ethics Committee (Ethics Committee on Animal Experimentation/UFPI, No. 274/16, approved on 16 September 2016) and adhered to the current guidelines for the care and use of laboratory animals, as well as the ethical standards for conducting research on experimental pain in conscious animals [29]. Female rats and male mice were used in separate experimental protocols, according to the established procedures of our research group. No direct comparisons between species or sexes were performed.

### 2.6. CFA-Induced Inflammation

The method used was based on the description by Auh and Ro [30]. Female Wistar rats received an intraplantar injection of 50 μL of Complete Freund’s adjuvant (CFA; 0.5 mg/mL of *Mycobacterium butyricum*, Difco^®^) on the plantar surface of the right hind paw. Animals in the control (sham) group received an injection of the same volume of 0.9% saline solution following the same procedure. At different time points following the CFA injection, the following parameters were assessed:

#### 2.6.1. Mechanical Hyperalgesia

Mechanical hyperalgesia was assessed using the modified Randall–Selitto paw pressure test [31]. Paw withdrawal thresholds were measured with an algesymeter (Insight Equipamentos, Ribeirão Preto, SP, Brazil). An increasing weight was applied to each paw until a withdrawal reflex was elicited. The investigator was trained to apply the tip perpendicularly to the central area of the hind paw, gradually increasing the pressure. The endpoint was defined as the removal of the paw accompanied by clear movements. Following withdrawal, the intensity of the applied pressure was recorded automatically.

Animals were tested before and after treatment, with three consecutive measurements taken at each time point and the thresholds averaged for statistical analysis. Twenty-four hours after CFA injection, the rats received oral treatment with α-PHEL at doses of 6.25, 12.5, 25, 50, and 100 mg/kg, α-PHEL/β-CD at doses of 3.12, 6.25, and 12.5 mg/kg, vehicle (2% Tween 80 in 0.9% saline or β-CD at 12.5 mg/kg), or diclofenac at 5 mg/kg. The α-PHEL dose range was selected based on previous studies investigating its antinociceptive and anti-inflammatory effects [23]. Based on the loading efficiency of the α-PHEL/β-CD preparation, the tested preparation doses of 3.12, 6.25, and 12.5 mg/kg corresponded to α-PHEL contents of 0.218, 0.438, and 0.875 mg/kg, respectively.

The withdrawal threshold was assessed before CFA injection (baseline) to obtain data strictly related to the treatments. Mechanical hyperalgesia was examined immediately before treatment (0 h) and at 1, 2, 3, 4, 5, and 6 h post-treatment (acute phase). To evaluate the effects of chronic treatment with α-PHEL and α-PHEL/β-CD, animals were treated and assessed once daily for 10 days (chronic phase).

#### 2.6.2. Evaluation of CFA-Induced Allodynia

To evaluate the antiallodynic effects of free α-PHEL and the α-PHEL/β-CD preparation, the digital von Frey method was employed. This method utilizes a digital von Frey apparatus (AVS Projetos, Campinas, SP, Brazil), equipped with a force transducer and a polypropylene tip, to apply gradually increasing pressure to the right hind paw of the animal, allowing for the determination of mechanical allodynia [32]. As with the assessment of hyperalgesia, the investigator was trained to apply the polypropylene tip to the central region of the right hind paw, with withdrawal movements recorded immediately.

Evaluations were conducted both before and after treatment, with three consecutive measurements taken to calculate the mean values for subsequent statistical analysis. Twenty-four hours following CFA injection, the rats received oral treatment with α-PHEL at doses of 12.5, 25, 50, and 100 mg/kg, α-PHEL/β-CD at doses of 3.12, 6.25, and 12.5 mg/kg, vehicle (2% Tween 80 in 0.9% saline or β-CD at 12.5 mg/kg), or dexamethasone at 0.5 mg/kg. The 6.25 mg/kg dose of α-PHEL was not included in the mechanical allodynia protocol because it did not produce a significant effect in the initial mechanical hyperalgesia evaluation.

The withdrawal threshold was assessed prior to CFA injection (baseline) to ensure that data reflected the effects of the treatments alone. Mechanical allodynia was examined immediately before treatment (0 h) and at 1, 2, 4, 6, 8, 10, 12, and 24 h post-treatment (acute phase). To investigate the chronic effects of α-PHEL and α-PHEL/β-CD, animals were treated and assessed once daily for a duration of 10 days (chronic phase).

For the chronic protocol, animals were treated and evaluated once daily for five consecutive days. Treatment was then interrupted for three days (days 6–8) to investigate the possible development of tolerance and resumed on days 9 and 10. Mechanical withdrawal thresholds were evaluated daily throughout the experimental period.

### 2.7. Investigation of the Participation of Opioid Receptors in the Antihypernociceptive Effect of α-PHEL

To evaluate the involvement of opioid receptors in the antinociceptive effect of α-PHEL, mechanical hypersensitivity was induced by the intraplantar administration of CFA (50 μL/paw) into the right hind paw of female Wistar rats. Twenty-four hours after CFA administration, the animals were allocated to the following groups: vehicle, morphine (5 mg/kg, i.p.), naloxone (3 mg/kg, i.p.), α-PHEL (100 mg/kg, p.o.), naloxone plus morphine, and naloxone plus α-PHEL. Naloxone, a non-selective opioid receptor antagonist, was administered 30 min before morphine or α-PHEL [33]. The 100 mg/kg dose of α-PHEL was selected because it produced the best antinociceptive response, allowing for the detection of a possible reduction or reversal of its effect following pharmacological pretreatment. Mechanical allodynia was assessed using the electronic von Frey test 30 min after morphine administration or 1 h after α-PHEL administration.

### 2.8. Investigation of the Serotonergic System in the Antihypernociceptive Effect of α-PHEL

The involvement of the serotonergic system was investigated using two complementary protocols. The first protocol evaluated the contribution of serotonin synthesis, whereas the second evaluated the involvement of 5-HT_2_A receptors.

In the first protocol, animals were distributed into four experimental groups: (1) vehicle, (2) PCPA + vehicle, (3) vehicle + α-PHEL, and (4) PCPA + α-PHEL. Serotonin depletion was induced by the administration of PCPA (100 mg/kg, i.p.), a tryptophan hydroxylase inhibitor, once daily for four consecutive days. On the third day of treatment, CFA (50 μL/paw) was injected into the right hind paw of female Wistar rats to induce mechanical hypersensitivity. On the fourth day, 30 min after the last administration of PCPA or vehicle, animals received α-PHEL (100 mg/kg, p.o.) or vehicle. Mechanical allodynia was evaluated 1 h later using the electronic von Frey test.

In the second protocol, animals were allocated to four groups: (1) vehicle, (2) ketanserin + vehicle, (3) vehicle + α-PHEL, and (4) ketanserin + α-PHEL. Mechanical hypersensitivity was induced by intraplantar injection of CFA (50 μL/paw). Twenty-four hours later, animals received ketanserin (0.3 mg/kg, i.p.), a 5-HT_2_A receptor antagonist, or its corresponding vehicle. After 20 min, α-PHEL (100 mg/kg, p.o.) or vehicle was administered, and mechanical allodynia was assessed 1 h later using the electronic von Frey test [34].

### 2.9. Measurement of Motor Performance and Locomotor Activity

The open-field and rotarod tests were performed to determine whether the treatments caused locomotor or motor coordination alterations that could interfere with the behavioral responses observed in the nociceptive tests.

#### 2.9.1. Open Field Test

The open-field test was conducted using an acrylic chamber with transparent walls measuring 30 cm × 30 cm × 15 cm, with the floor divided into nine equal squares. Male Swiss mice were placed in the chamber one day before the experiment for acclimatization. The animals received α-PHEL (100 mg/kg, p.o.), α-PHEL/β-CD (12.5 mg/kg, p.o.), vehicle (10 mL/kg, p.o.), or diazepam (4 mg/kg, i.p.). α-PHEL, α-PHEL/β-CD, and vehicle were administered 1 h before the test, whereas diazepam was administered 30 min before the test. During a 5 min observation period, the number of crossings, defined as the movement of all four paws into a new square, was recorded [35].

#### 2.9.2. Rotarod Test

The rotarod apparatus (model RR-2002, Insight Equipamentos, Ribeirão Preto, SP, Brazil) consisted of a rotating rod with a diameter of 2.5 cm, subdivided into four compartments by 25 cm diameter disks. Male Swiss mice were trained on the apparatus one day before the experiment, and animals capable of remaining on the rotating rod for 60 s were included in the test. The mice received α-PHEL (100 mg/kg, p.o.), α-PHEL/β-CD (12.5 mg/kg, p.o.), vehicle (10 mL/kg, p.o.), or diazepam (4 mg/kg, i.p.). α-PHEL, α-PHEL/β-CD, and vehicle were administered 1 h before the test, whereas diazepam was administered 30 min before the test. Each animal was placed on the rod rotating at 12 rpm, and the time spent on the rod was recorded, with a cutoff time of 60 s [36].

### 2.10. Statistical Analysis

Results are expressed as mean ± standard error of the mean (SEM). Data from the mechanical hyperalgesia and allodynia experiments were analyzed using two-way repeated-measures ANOVA followed by Bonferroni’s multiple comparisons test. The Greenhouse–Geisser correction was applied when necessary. Data from the other experiments were analyzed using one-way ANOVA followed by Tukey’s or Bonferroni’s multiple comparisons test, as appropriate. Normality and homogeneity of variance were assessed before analysis. Differences were considered significant when *p* < 0.05. Statistical analyses were performed using GraphPad Prism version 9.00 for Windows (GraphPad Software, San Diego, CA, USA).

## 3. Results

### 3.1. Obtaining and Characterization of the α-PHEL/β-CD Preparation

#### 3.1.1. Encapsulation Efficiency and Loading Efficiency

According to the calculations, the α-PHEL/β-CD preparation showed an encapsulation efficiency of 70.7 ± 1.5% and a loading efficiency of 7.0 ± 0.2% (Table 1). These results indicate that approximately 70% of the initial α-PHEL was recovered from the β-CD matrix following extraction. Accordingly, approximately 7 mg of α-PHEL was recovered per 100 mg of the spray-dried α-PHEL/β-CD preparation.

Table 2 presents the amount of α-PHEL contained in the doses of the α-PHEL/β-CD preparation used in the experimental protocols, calculated proportionally based on the established loading efficiency.

#### 3.1.2. Molecular Modeling

**Figure 1:**
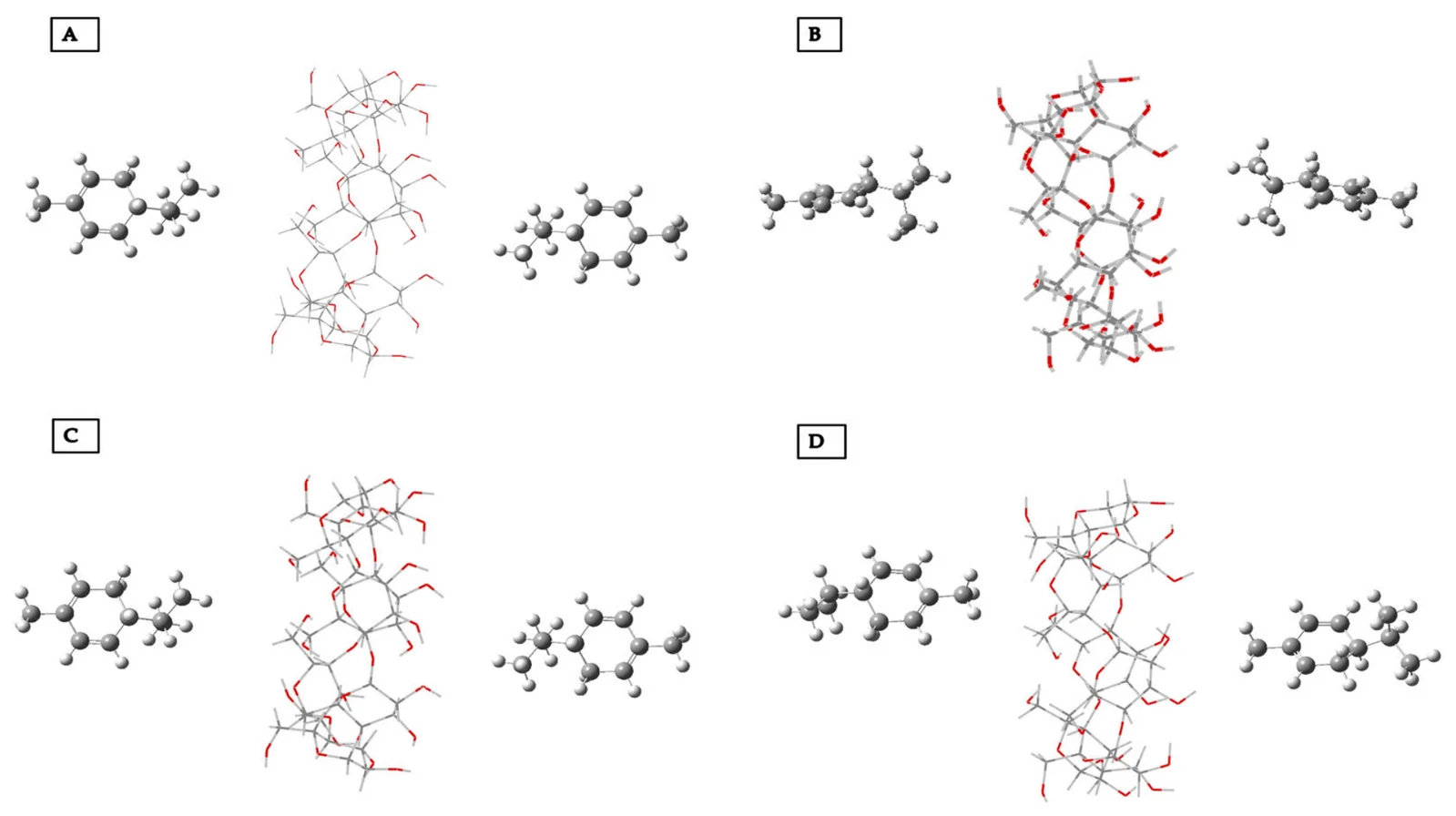
Initial orientations adopted for α-PHEL relative to the β-cyclodextrin cavity before PM6 geometry optimization. Panels (**A**–**D**) represent the four distinct starting orientations used for geometry optimization. Atom colors: carbon, gray; oxygen, red; hydrogen, white.

**Figure 2:**
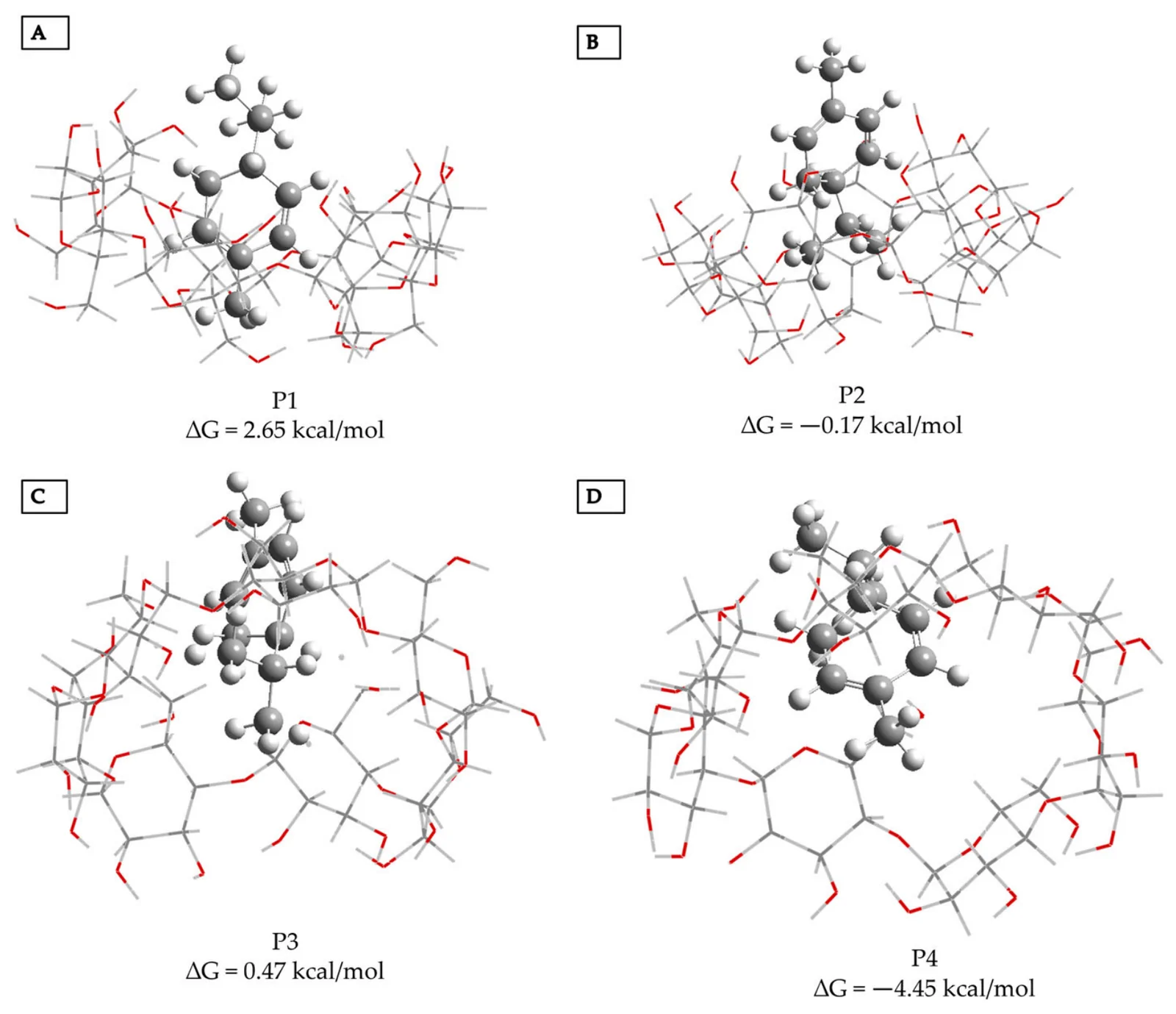
Optimized theoretical α-PHEL/β-CD host–guest configurations obtained using the PM6 method. Panels (**A**–**D**) correspond to the optimized orientations P1–P4, respectively. Calculated Gibbs free energy (ΔG) values are indicated below each configuration. Orientation P4 showed the lowest calculated Gibbs free energy (ΔG = −4.45 kcal/mol), suggesting that it was the most energetically favorable among the evaluated orientations under the conditions of the computational model. Atom colors: carbon, gray; oxygen, red; hydrogen, white. α-PHEL is shown in ball-and-stick representation, whereas β-CD is shown in wireframe representation.

Four distinct initial orientations of α-PHEL relative to the β-CD cavity were generated before geometry optimization to evaluate the influence of guest orientation on the theoretical stability of the host–guest system (Figure 1). After optimization, the corresponding theoretical host–guest configurations were obtained, and their calculated Gibbs free energies are summarized in Table 3. Among the evaluated configurations, orientation P4 showed the lowest calculated Gibbs free energy, suggesting that it was the most energetically favorable orientation under the conditions of the computational model (Figure 2).

The greater theoretical stability observed for orientations P2 and P4 may be related to more favorable van der Waals and hydrophobic interactions between α-PHEL and the β-CD cavity, suggesting possible insertion through the narrower side of β-CD.

#### 3.1.3. Infrared Absorption Spectroscopy

**Figure 3:**
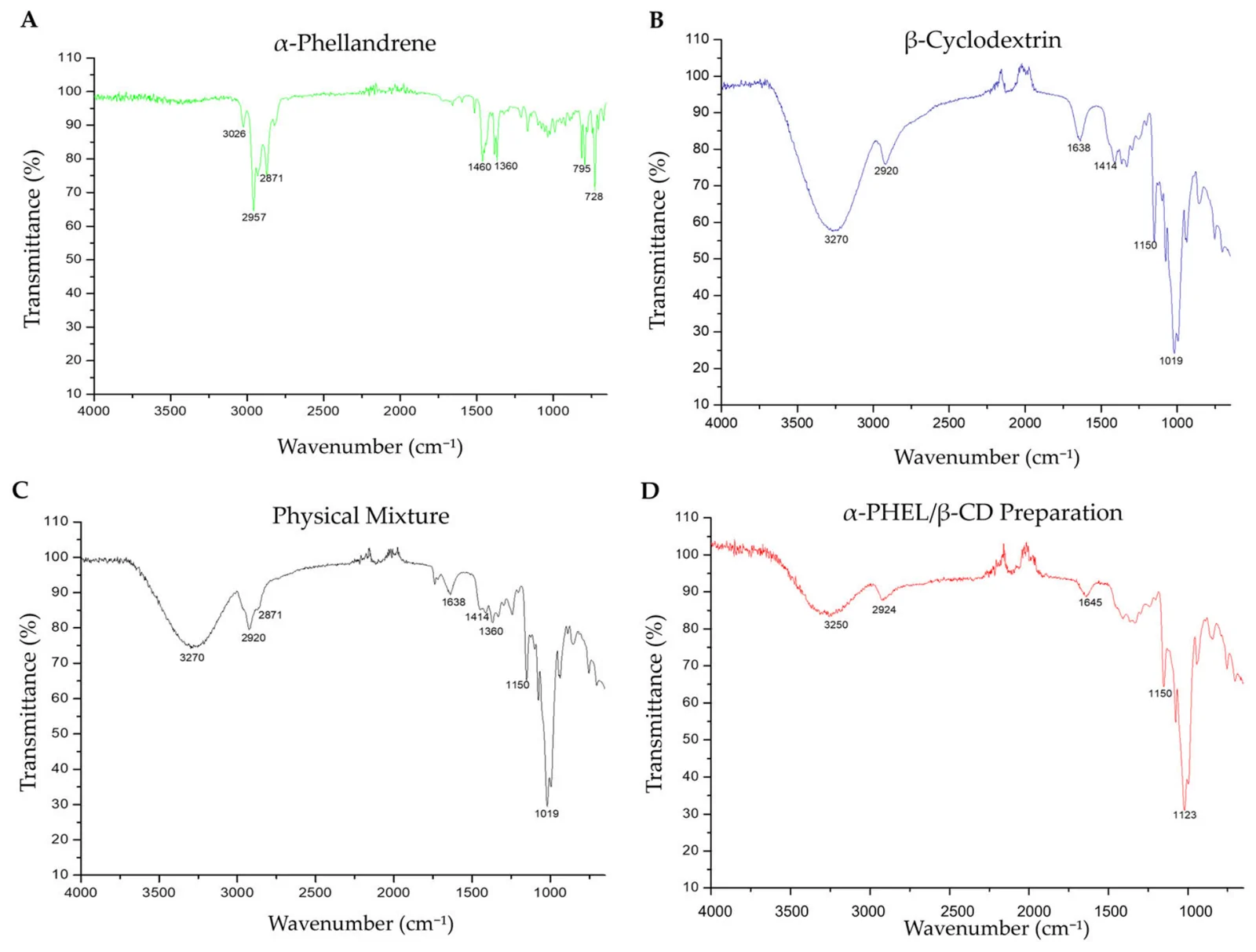
FTIR spectra of (**A**) α-phellandrene (α-PHEL), (**B**) β-cyclodextrin (β-CD), (**C**) the physical mixture (α-PHEL + β-CD), and (**D**) the spray-dried α-PHEL/β-CD preparation. The reduction in the intensity of characteristic α-PHEL absorption bands and the shifts observed in the O–H and C–H stretching regions are consistent with interactions between α-PHEL and β-CD and suggest possible host–guest inclusion.

The infrared (IR) absorption spectrum of α-PHEL (Figure 3A) exhibited characteristic peaks at 2871 cm^−1^, 2957 cm^−1^, and 3026 cm^−1^, corresponding to C–H stretching vibrations. A peak at 1460 cm^−1^ was also observed, associated with CH_2_ and C=C stretching vibrations, characteristic of hydrocarbon structures.

The IR spectrum of β-CD (Figure 3B) showed absorption bands at 3270 cm^−1^ (O–H stretch), 2920 cm^−1^ (C–H stretch), 1150 cm^−1^ (C–O stretch), and 1019 cm^−1^ (O–C–O stretch). The physical mixture of α-PHEL and β-CD (Figure 3C) displayed characteristic peaks from both components, suggesting minimal or no interaction between them.

In contrast, the spectrum of the spray-dried α-PHEL/β-CD preparation (Figure 3D) presented bands predominantly corresponding to β-CD, with a notable reduction in the intensity of the α-PHEL characteristic peaks. Additionally, shifts were observed in the O–H stretching band (from 3270 cm^−1^ to 3250 cm^−1^) and in the C–H stretching band (from 2920 cm^−1^ to 2924 cm^−1^).

These spectral changes indicate modifications in the molecular environment of α-PHEL in the presence of β-CD and suggest possible host–guest inclusion.

### 3.2. Effect of α-PHEL on CFA-Induced Mechanical Hyperalgesia

**Figure 4:**
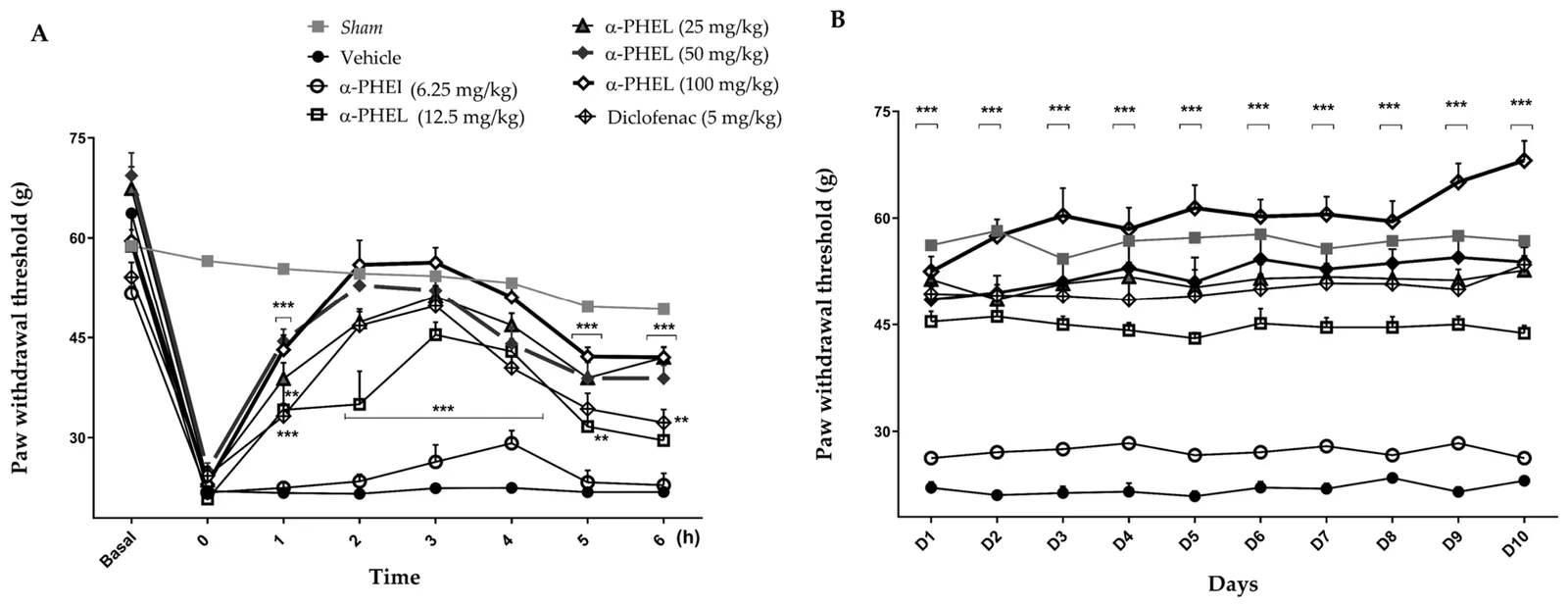
Effect of acute (**A**) and chronic (**B**) administration of α-phellandrene (α-PHEL) on CFA-induced mechanical hyperalgesia in female Wistar rats evaluated using the modified Randall–Selitto test. Mechanical hyperalgesia was induced by intraplantar injection of CFA (50 μL/paw). Data are expressed as mean ± SEM (*n* = 6–8 rats per group). Statistical analysis was performed using two-way repeated-measures ANOVA followed by Bonferroni’s multiple comparisons test, with the Greenhouse–Geisser correction when necessary. ** *p* < 0.01 and *** *p* < 0.001 versus the CFA + vehicle group at the corresponding experimental time point (**A**) or experimental day (**B**).

The intraplantar injection of CFA in female Wistar rats resulted in significant mechanical hyperalgesia 24 h post-injection, which persisted throughout the study period. Administration of α-PHEL at doses of 12.5, 25, 50, and 100 mg/kg (p.o.) produced a substantial reduction in mechanical hyperalgesia, observed as early as 1 h post-treatment compared to the vehicle group. The antihyperalgesic effect varied according to dose and time, with the peak effect observed at three hours post-administration. At this time point, the mean paw withdrawal threshold was 22.44 ± 1.02 g in the CFA + vehicle group, whereas α-PHEL treatment increased the thresholds to 45.4 ± 1.8, 51.2 ± 2.4, 52.1 ± 1.6, and 56.3 ± 2.2 g at doses of 12.5, 25, 50, and 100 mg/kg, respectively (*p* < 0.001 vs. CFA + vehicle; Figure 4A).

**Figure 5:**
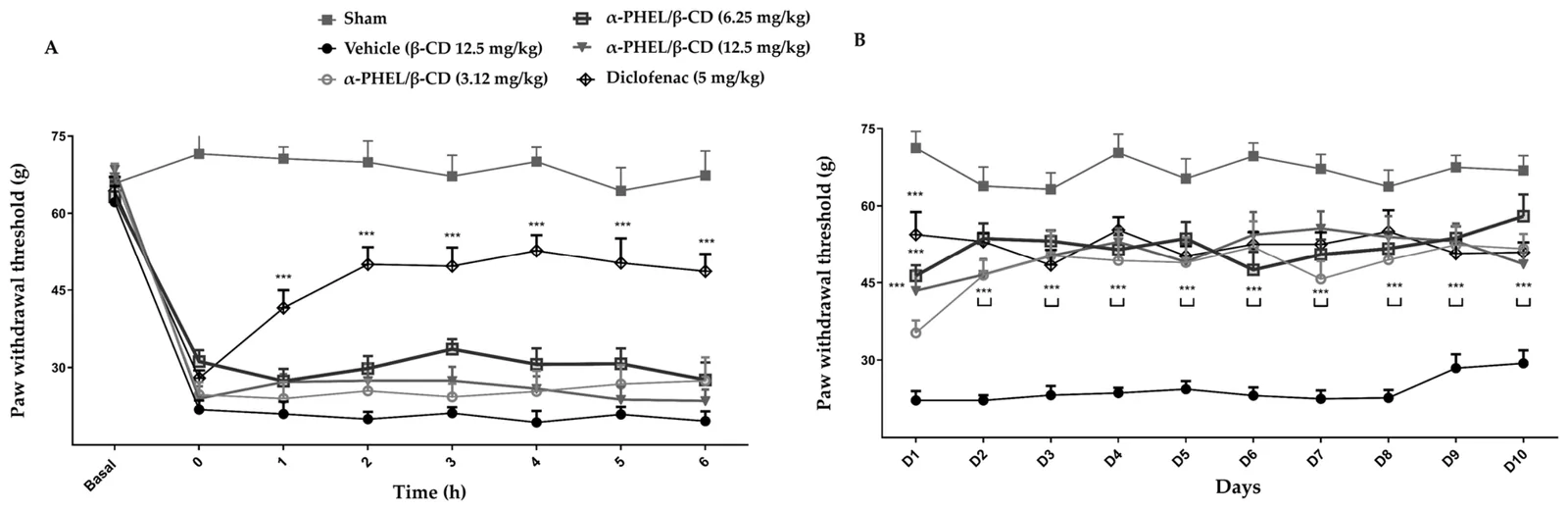
Effect of acute (**A**) and chronic (**B**) administration of the α-PHEL/β-CD preparation on CFA-induced mechanical hyperalgesia in female Wistar rats evaluated using the modified Randall–Selitto test. Mechanical hyperalgesia was induced by intraplantar injection of CFA (50 μL/paw). Data are expressed as mean ± SEM (*n* = 6–8 rats per group). Statistical analysis was performed using two-way repeated-measures ANOVA followed by Bonferroni’s multiple comparisons test, with the Greenhouse–Geisser correction when necessary. Statistical significance is indicated at each corresponding experimental time point (**A**) or experimental day (**B**). *** *p* < 0.001 versus the β-CD group.

In contrast, acute administration of the α-PHEL/β-CD preparation at doses of 3.12, 6.25, and 12.5 mg/kg (p.o.) did not significantly increase the mechanical withdrawal threshold compared with the β-CD group. At 3 h post-treatment, the mean paw withdrawal threshold was 21.19 ± 1.15 g in the β-CD group and 24.31 ± 2.54, 33.58 ± 1.95, and 27.44 ± 2.67 g in the groups treated with α-PHEL/β-CD at doses of 3.12, 6.25, and 12.5 mg/kg, respectively (*p* > 0.05 vs. β-CD; Figure 5A).

During chronic treatment, daily administration of α-PHEL over ten consecutive days resulted in a significant reduction in CFA-induced mechanical hyperalgesia. On day 10, the mean paw withdrawal threshold in the CFA + vehicle group was 23.09 ± 0.60 g, whereas α-PHEL treatment increased the withdrawal thresholds to 43.75 ± 1.07, 52.67 ± 2.03, 53.83 ± 2.10, and 68.10 ± 2.76 g at doses of 12.5, 25, 50, and 100 mg/kg, respectively (*p* < 0.001 vs. CFA + vehicle; Figure 4B).

In contrast to the acute treatment, all evaluated doses of α-PHEL/β-CD (3.12, 6.25, and 12.5 mg/kg, p.o.) significantly reduced mechanical hyperalgesia during the chronic treatment phase. On day 10, the mean paw withdrawal threshold was 29.38 ± 2.51 g in the β-CD group, whereas treatment with α-PHEL/β-CD increased the withdrawal thresholds to 51.65 ± 2.88, 58.00 ± 4.22, and 48.64 ± 2.26 g at doses of 3.12, 6.25, and 12.5 mg/kg, respectively (*p* < 0.0001, *p* < 0.0001, and *p* < 0.001 vs. β-CD, respectively; Figure 5B).

### 3.3. Effect of α-PHEL on CFA-Induced Mechanical Allodynia

**Figure 6:**
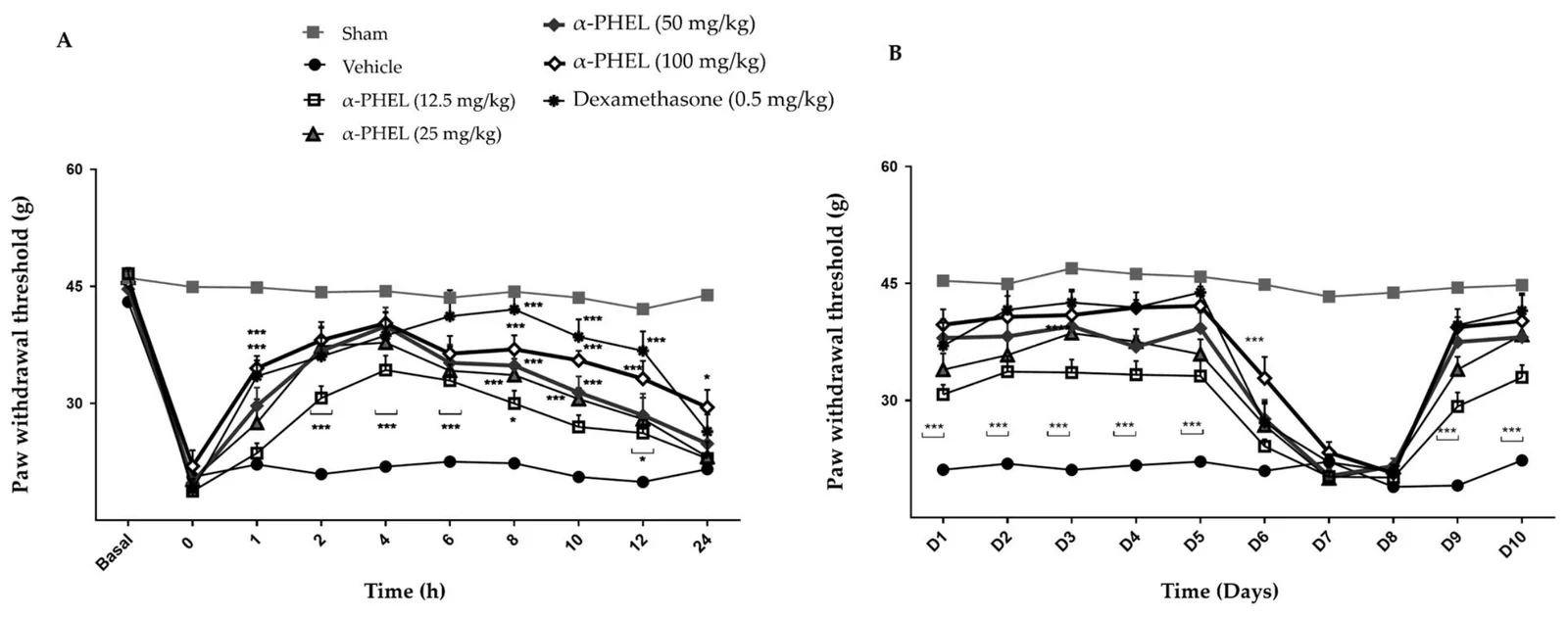
Effect of acute (**A**) and chronic (**B**) administration of α-PHEL on CFA-induced mechanical allodynia in female Wistar rats evaluated using the electronic von Frey test. Mechanical allodynia was induced by intraplantar injection of CFA (50 μL/paw). Data are expressed as mean ± SEM (*n* = 6–8 rats per group). Statistical analysis was performed using two-way repeated-measures ANOVA followed by Bonferroni’s multiple comparisons test, with the Greenhouse–Geisser correction when necessary. Statistical significance is indicated at each corresponding experimental time point (**A**) or experimental day (**B**). * *p* < 0.05, *** *p* < 0.001, and **** *p* < 0.0001 versus the CFA + vehicle group.

A significant decrease in the mechanical withdrawal threshold, indicative of mechanical allodynia, was observed 24 h after CFA administration (50 μL/paw). This reduction persisted throughout the experimental period compared to baseline values and the sham group. A single dose of α-PHEL (100 mg/kg; Figure 6A) significantly decreased CFA-induced mechanical allodynia during the first hour (*** *p* < 0.001), with sustained effects up to 24 h (*** *p* < 0.001). Treatment with α-PHEL at doses of 25 and 50 mg/kg significantly alleviated mechanical allodynia from the second to the twelfth hour post-treatment, while the 12.5 mg/kg dose showed a significant reduction from the second hour (*** *p* < 0.001) to the eighth hour (* *p* < 0.05), with maximal inhibition at the fourth hour (Figure 6A).

At this time point, the mean paw withdrawal threshold in the CFA + vehicle group was 21.90 ± 1.12 g, whereas α-PHEL treatment increased the thresholds to 34.3 ± 1.8, 37.7 ± 2.1, 39.8 ± 2.4, and 40.3 ± 1.4 g at doses of 12.5, 25, 50, and 100 mg/kg, respectively (*p* < 0.0001 vs. CFA + vehicle; Figure 6A).

**Figure 7:**
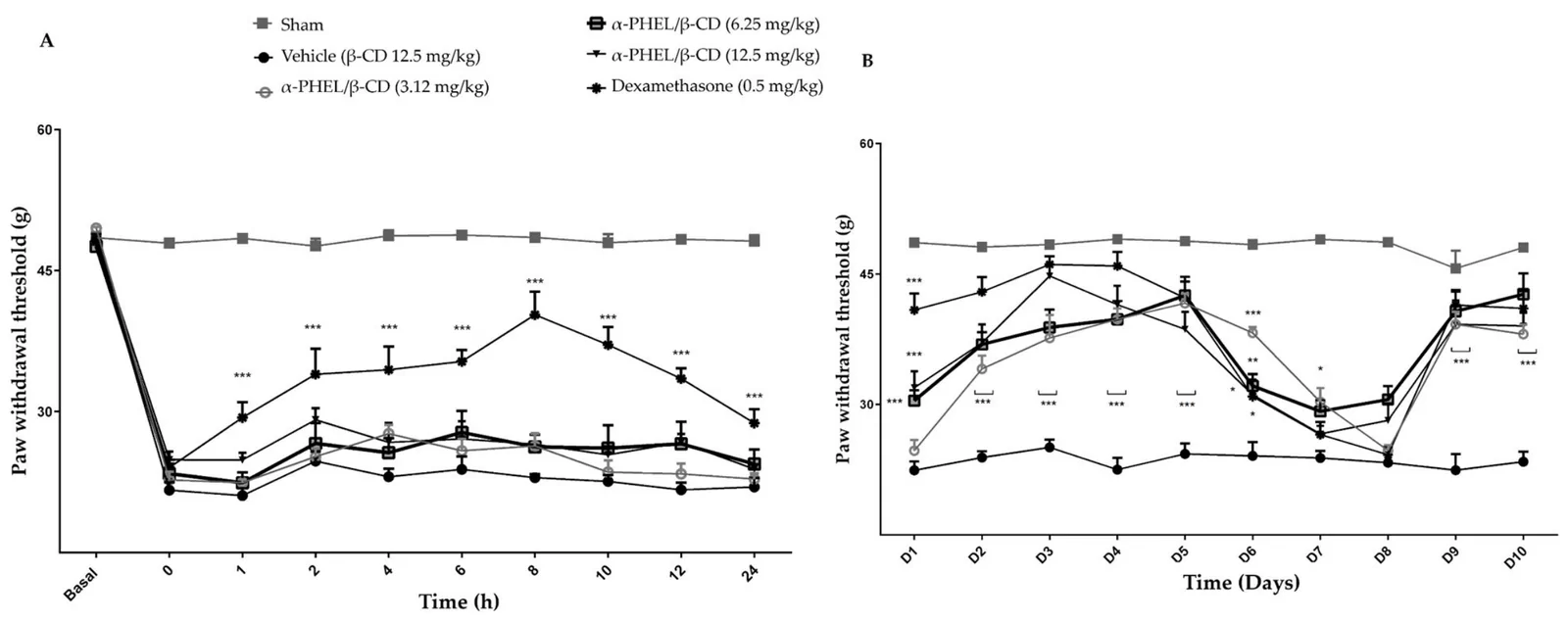
Effect of acute (**A**) and repeated (**B**) administration of the α-PHEL/β-CD preparation on CFA-induced mechanical allodynia in female Wistar rats evaluated using the electronic von Frey test. CFA (50 μL/paw) was administered intraplantarly, and dexamethasone (0.5 mg/kg, p.o.) was used as the positive control. Data are expressed as mean ± SEM (*n* = 6–8 rats per group) and were analyzed using two-way repeated-measures ANOVA with the Greenhouse–Geisser correction when necessary, followed by Bonferroni’s multiple comparisons tests. * *p* < 0.05, ** *p* < 0.01, *** *p* < 0.001 versus the β-CD group at the corresponding time point (**A**) or experimental day (**B**).

In contrast, the α-PHEL/β-CD preparation (3.12, 6.25, and 12.5 mg/kg, p.o.) did not demonstrate a significant effect on mechanical allodynia after a single dose. The maximum mean paw withdrawal thresholds were 27.66 ± 0.93 g at 4 h for 3.12 mg/kg, 27.79 ± 2.29 g at 6 h for 6.25 mg/kg, and 29.13 ± 1.24 g at 2 h for 12.5 mg/kg. The corresponding values in the β-CD group were 23.06 ± 0.86, 23.85 ± 1.39, and 24.73 ± 1.01 g, respectively (*p* > 0.05 vs. β-CD; Figure 7A).

During prolonged treatment, daily administration of α-PHEL (12.5, 25, 50, and 100 mg/kg, p.o.) significantly reduced CFA-induced mechanical allodynia. The greatest antiallodynic response was observed with α-PHEL at 100 mg/kg, which reached a paw withdrawal threshold of 42.1 ± 2.4 g on day 5. The 12.5, 25, and 50 mg/kg doses also produced significant effects, reaching withdrawal thresholds of 33.5 ± 1.7, 38.6 ± 1.2, and 39.5 ± 2.3 g, respectively, on day 3 (*p* < 0.001 vs. CFA + vehicle; Figure 6B).

Similarly, all doses of the α-PHEL/β-CD preparation significantly reduced mechanical allodynia during prolonged treatment. The 12.5 mg/kg dose showed its maximum effect on day 3, reaching a paw withdrawal threshold of 44.76 ± 1.45 g, compared with 25.06 ± 0.88 g in the β-CD group. The 3.12 and 6.25 mg/kg doses reached their maximal effects on day 5, with withdrawal thresholds of 41.64 ± 1.11 and 42.53 ± 2.16 g, respectively, compared with 24.31 ± 1.21 g in the β-CD group (*p* < 0.0001 for all comparisons; Figure 7B).

Following a three-day treatment interruption, mechanical allodynia was gradually reestablished. However, α-PHEL at 100 mg/kg and all doses of α-PHEL/β-CD maintained antiallodynic effects on the first day after treatment cessation. On day 6, the mean paw withdrawal threshold in the β-CD group was 24.09 ± 1.58 g, whereas the thresholds in the groups previously treated with α-PHEL/β-CD at 3.12, 6.25, and 12.5 mg/kg were 38.23 ± 0.68, 32.14 ± 1.33, and 31.11 ± 1.76 g, respectively (*p* < 0.0001, *p* < 0.001, and *p* < 0.01 vs. β-CD, respectively; Figure 7B). On day 7, only the 3.12 mg/kg dose remained significantly different from the β-CD group (30.30 ± 1.58 vs. 23.85 ± 0.79 g; *p* < 0.05), whereas on day 8 only the 6.25 mg/kg dose remained significant (30.56 ± 1.54 vs. 23.30 ± 0.90 g; *p* < 0.01 vs. β-CD).

Upon resuming treatment on day 9, α-PHEL and the α-PHEL/β-CD preparation effectively reduced mechanical allodynia. The paw withdrawal threshold in the β-CD group was 22.44 ± 1.85 g, whereas α-PHEL/β-CD at 3.12, 6.25, and 12.5 mg/kg increased the thresholds to 39.23 ± 1.42, 40.70 ± 2.28, and 39.21 ± 2.76 g, respectively (*p* < 0.0001 vs. β-CD for all comparisons; Figure 7B). These effects were maintained until the end of the study period. On day 10, the corresponding thresholds were 38.09 ± 1.09, 42.68 ± 2.39, and 39.03 ± 1.52 g, compared with 23.43 ± 1.15 g in the β-CD group (*p* < 0.0001 vs. β-CD for all comparisons; Figure 7B).

### 3.4. Investigating the Involvement of the Opioidergic System in the Antihypernociceptive Effect of α-PHEL

**Figure 8:**
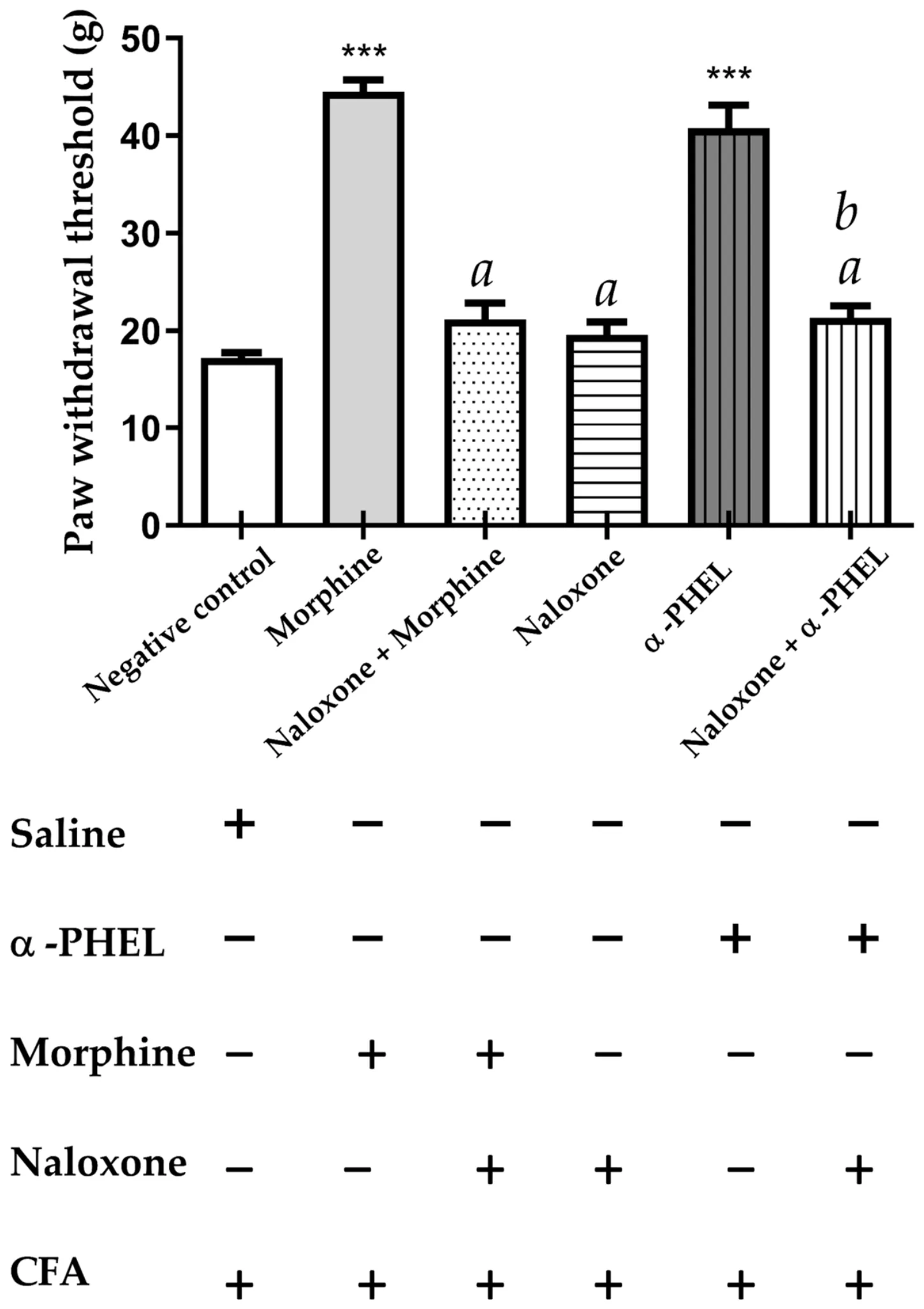
Effect of naloxone on the antihypernociceptive activity of α-PHEL (100 mg/kg, p.o.) in CFA-induced mechanical hyperalgesia in female Wistar rats. Mechanical hyperalgesia was induced by intraplantar injection of CFA (50 μL/paw) and evaluated using the modified Randall–Selitto test. α-PHEL (100 mg/kg, p.o.), morphine (5 mg/kg, i.p.) and naloxone (3 mg/kg, i.p.) were administered according to the experimental protocol. Data are expressed as mean ± SEM (*n* = 6–8 rats per group). Statistical analysis was performed using one-way ANOVA followed by Bonferroni’s multiple comparisons test. *** *p* < 0.001 versus the CFA + vehicle group; *a p* < 0.001 versus the morphine-treated group; *b p* < 0.001 versus the α-PHEL-treated group.

The results presented in Figure 8 indicate that naloxone alone did not exert an antinociceptive effect, with a paw withdrawal threshold of 19.60 ± 1.27 g, comparable to that observed in the CFA + vehicle group (17.22 ± 0.54 g). In contrast, morphine (5 mg/kg, i.p.) increased the paw withdrawal threshold to 44.53 ± 1.21 g, whereas naloxone pretreatment reduced this response to 21.20 ± 1.66 g, corresponding to a 47.6% reversal of the antinociceptive effect of morphine and confirming the expected pharmacological activity of naloxone.

Similarly, pretreatment with naloxone significantly reduced the antihypernociceptive effect of α-PHEL (100 mg/kg, p.o.). α-PHEL alone increased the paw withdrawal threshold to 40.75 ± 2.39 g, whereas naloxone pretreatment reduced this response to 21.33 ± 1.22 g, corresponding to a 52.3% reversal of the antihypernociceptive effect of α-PHEL. These findings indicate that opioid signaling contributes, at least in part, to the antihypernociceptive effect of α-PHEL under the present experimental conditions.

### 3.5. Investigating the Involvement of the Serotonergic System in the Antihypernociceptive Effect of α-PHEL

**Figure 9:**
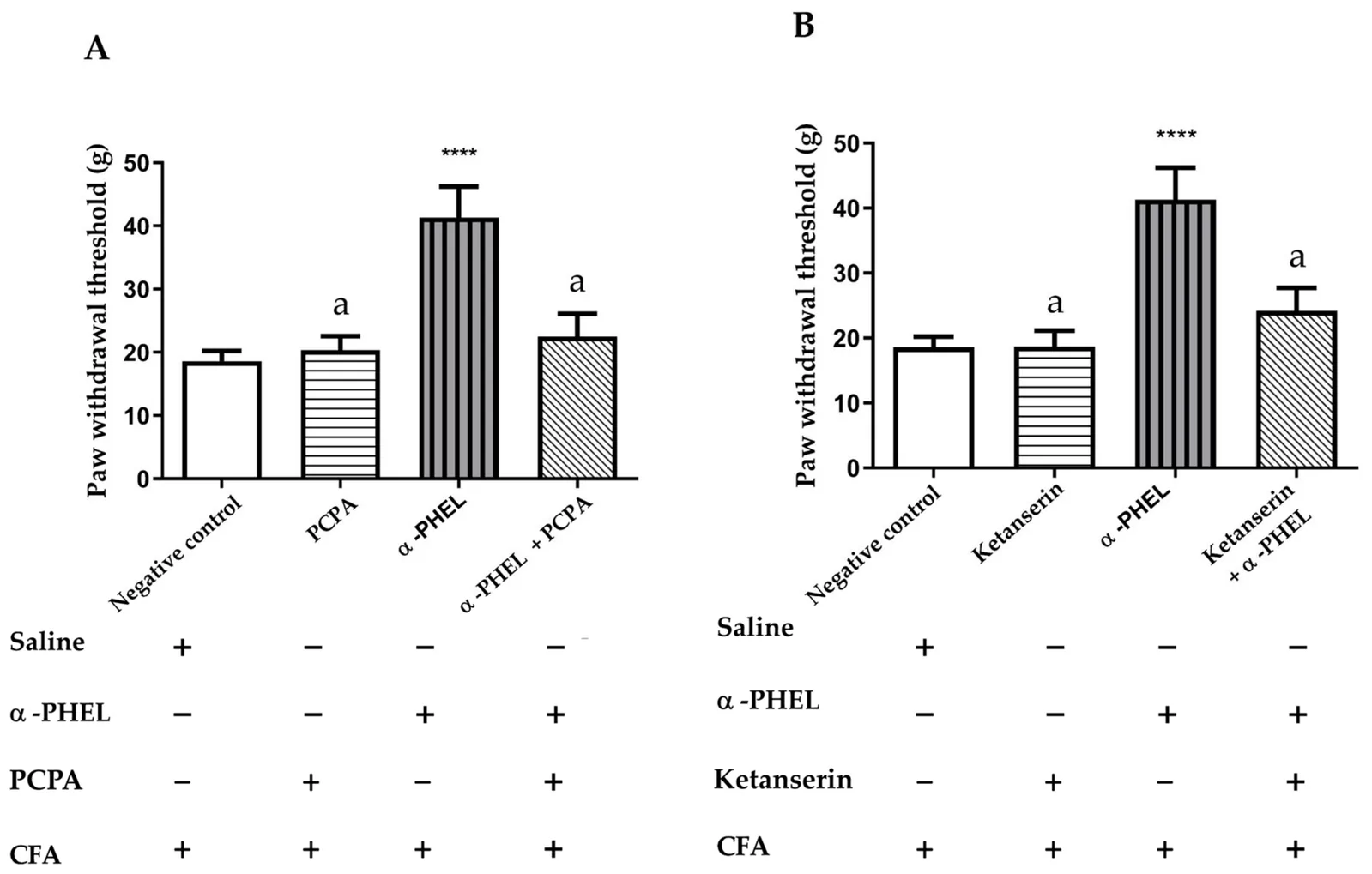
Involvement of the serotonergic system in the antinociceptive effect of α-PHEL (100 mg/kg, p.o.) in CFA-induced mechanical allodynia in female Wistar rats. (**A**) Effect of pretreatment with p-chlorophenylalanine (PCPA, 100 mg/kg, i.p.). (**B**) Effect of pretreatment with ketanserin (0.3 mg/kg, i.p.). Mechanical allodynia was induced by intraplantar injection of CFA (50 μL/paw) and evaluated using the electronic von Frey test. Data are expressed as mean ± SEM (*n* = 6–8 rats per group). Statistical analysis was performed using one-way ANOVA followed by Bonferroni’s multiple comparisons test. **** *p* < 0.0001 versus the CFA + vehicle group; a *p* < 0.0001 versus the α-PHEL-treated group.

As shown in Figure 9A, α-PHEL (100 mg/kg, p.o.) significantly increased the paw withdrawal threshold to 41.32 ± 2.00 g compared with the CFA + vehicle group (18.60 ± 0.67 g; *p* < 0.0001). PCPA administered alone did not significantly alter the paw withdrawal threshold (20.37 ± 0.90 g) compared with the vehicle group. However, pretreatment with PCPA, a serotonin synthesis inhibitor, significantly reduced the antihypernociceptive effect of α-PHEL, decreasing the paw withdrawal threshold to 22.52 ± 1.46 g (*p* < 0.0001 vs. α-PHEL), corresponding to a 54.5% reversal of the effect observed with α-PHEL. The response in the PCPA + α-PHEL group did not differ significantly from that of the CFA + vehicle group.

Similarly, ketanserin administered alone did not significantly alter the paw withdrawal threshold (18.70 ± 0.99 g) compared with the CFA + vehicle group (18.60 ± 0.67 g) (Figure 9B). α-PHEL (100 mg/kg, p.o.) significantly increased the paw withdrawal threshold to 41.32 ± 2.00 g (*p* < 0.0001 vs. CFA + vehicle), whereas pretreatment with ketanserin significantly reduced this response to 24.20 ± 1.44 g (*p* < 0.0001 vs. α-PHEL), corresponding to a 54.2% reversal of the effect observed with α-PHEL. The response in the ketanserin + α-PHEL group did not differ significantly from that of the CFA + vehicle group.

### 3.6. Open-Field and Rotarod Tests

**Figure 10:**
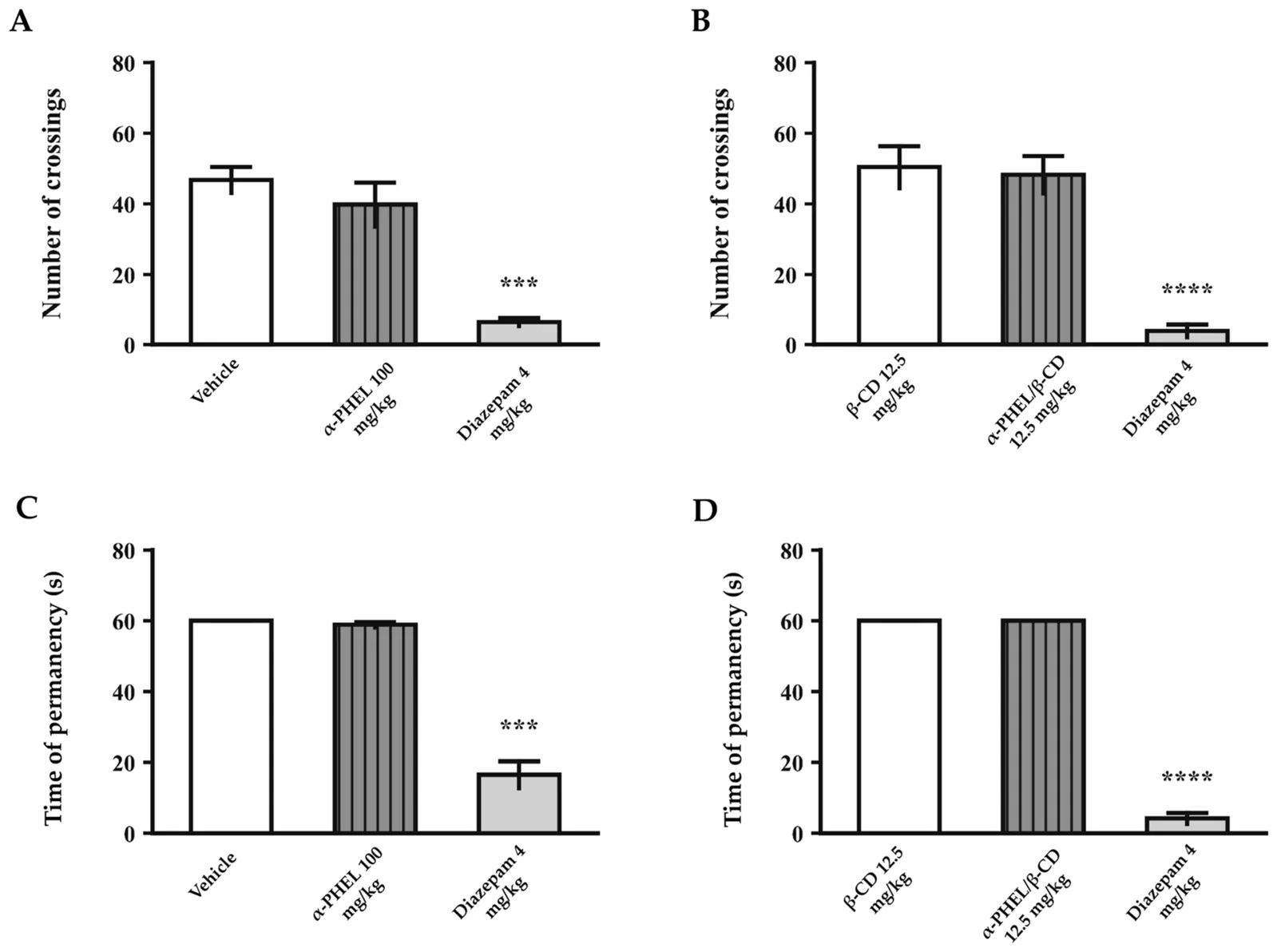
Locomotor activity was evaluated using the open-field test after treatment with α-PHEL (100 mg/kg, p.o.) (**A**) or α-PHEL/β-CD (12.5 mg/kg, p.o.) (**B**). Motor performance was evaluated using the rotarod test after treatment with α-PHEL (100 mg/kg, p.o.) (**C**) or α-PHEL/β-CD (12.5 mg/kg, p.o.) (**D**). Diazepam (4 mg/kg, i.p.) was used as the positive control. Data are expressed as mean ± SEM (*n* = 6–8 mice per group). Statistical analysis was performed using one-way ANOVA followed by Tukey’s test (**A**,**C**) or Bonferroni’s test (**B**,**D**). *** *p* < 0.001 and **** *p* < 0.0001 versus the corresponding vehicle group.

In the open-field test, α-PHEL at the highest dose tested (100 mg/kg, p.o.) did not alter the spontaneous locomotor activity of the animals. The mean number of crossings was 39.80 ± 6.19 in the α-PHEL group and 46.75 ± 3.69 in the vehicle group (*p* > 0.05; Figure 10A). Similarly, the α-PHEL/β-CD preparation at the highest dose tested (12.5 mg/kg, p.o.) did not alter spontaneous locomotor activity. The mean number of crossings was 60.33 ± 6.57 in the α-PHEL/β-CD group and 63.00 ± 7.44 in the vehicle group (*p* > 0.05; Figure 10B). Diazepam (4 mg/kg, i.p.) significantly reduced the number of crossings to 6.38 ± 1.12 and 4.83 ± 2.27 in the α-PHEL and α-PHEL/β-CD experiments, respectively (*p* < 0.001 and *p* < 0.0001 versus the corresponding vehicle group).

In the rotarod test, α-PHEL (100 mg/kg, p.o.) did not significantly alter the time spent by the animals on the rotating rod compared with the vehicle group (58.86 ± 0.77 and 60.00 ± 0.00 s, respectively; *p* > 0.05; Figure 10C). Likewise, the α-PHEL/β-CD preparation (12.5 mg/kg, p.o.) did not alter the time spent on the rotating rod, with mean values of 60.00 ± 0.00 s in both the treated and vehicle groups (*p* > 0.05; Figure 10D). In contrast, diazepam significantly reduced the time spent on the rotating rod to 16.50 ± 3.81 and 4.17 ± 1.56 s in the α-PHEL and α-PHEL/β-CD experiments, respectively (*p* < 0.001 and *p* < 0.0001 versus the corresponding vehicle group). These results suggest that α-PHEL and the α-PHEL/β-CD preparation did not produce muscle-relaxant or central nervous system depressant effects under the experimental conditions used.

## 4. Discussion

Cyclodextrins have been extensively employed to enhance the pharmaceutical performance of poorly water-soluble compounds by increasing their solubility, improving chemical stability and promoting greater bioavailability of a wide variety of substances [37]. These properties make cyclodextrins promising carriers for volatile compounds such as α-PHEL.

The α-PHEL/β-CD preparation showed an encapsulation efficiency of 70.7 ± 1.5% and a loading efficiency of 7.0 ± 0.2%. These values indicate the amount of α-PHEL recovered from the β-CD matrix under the extraction and quantification conditions used and are consistent with those reported for other cyclodextrin-based preparations of volatile natural products [38].

The preliminary PM6 molecular modeling suggested that the theoretical stability of the α-PHEL/β-CD host–guest system was influenced by the orientation of α-PHEL relative to the β-CD cavity. Orientations P2 and P4 showed negative Gibbs free energy values, with P4 presenting the lowest calculated value among the evaluated configurations. These results may reflect more favorable van der Waals and hydrophobic interactions and possible insertion of α-PHEL through the narrower side of β-CD. Because the calculations were performed in vacuum using a semiempirical method, the results provide a comparative theoretical estimate and do not account for solvent effects.

Therefore, they should be interpreted as supportive information regarding possible host–guest orientations rather than definitive evidence of binding stability. This interpretation is consistent with previous studies indicating that the geometry and polarity of guest molecules influence the stability of cyclodextrin host–guest systems [39,40].

The FTIR results showed changes in the spectral profile of the spray-dried α-PHEL/β-CD preparation compared with free α-PHEL, β-CD, and the physical mixture. The decreased intensity of characteristic α-PHEL bands and the shifts observed in the O–H and C–H stretching regions indicate changes in the molecular environment following preparation and are consistent with possible host–guest inclusion. Similar spectral changes have been reported for β-CD inclusion complexes and have been associated with non-covalent interactions between guest molecules and the cyclodextrin cavity [41,42].

Intraplantar CFA injection is a well-established model of persistent inflammatory pain characterized by reduced nociceptive thresholds and mechanical hyperalgesia [43]. The release of inflammatory mediators, including cytokines, prostanoids, and nitric oxide, contributes to peripheral nociceptor sensitization [44,45,46], while persistent inflammation may also contribute to central sensitization [7,45,46]. In this model, α-PHEL reduced CFA-induced mechanical hyperalgesia during both acute and repeated treatment.

Although free α-PHEL produced significant antihyperalgesic effects after acute administration, the α-PHEL/β-CD preparation was not effective during this phase. In contrast, during repeated treatment, all doses of α-PHEL/β-CD significantly increased the mechanical withdrawal threshold, including doses containing substantially lower amounts of α-PHEL than those administered as the free compound. This finding suggests that association with β-CD may influence the pharmaceutical properties of α-PHEL. Previous studies have shown that complexation with β-CD can improve the solubility and stability of poorly water-soluble compounds and may also protect volatile compounds from degradation [37,38]. Similar improvements in pharmacological response have been reported for β-CD complexes containing citronellal, carvacrol, and terpene-rich essential oils [47,48,49].

The absence of a significant effect after a single administration, followed by antinociceptive activity during repeated treatment, may reflect time-dependent changes in α-PHEL release, absorption, stability, or systemic exposure. However, these possibilities remain hypothetical because pharmacokinetic and oral bioavailability parameters were not evaluated in the present study.

A similar pattern was observed for mechanical allodynia during repeated treatment. All doses of α-PHEL/β-CD produced significant antiallodynic effects, including the 3.12 mg/kg dose, which corresponds to 0.218 mg/kg of α-PHEL. In the experiments with free α-PHEL, the 6.25 mg/kg dose was ineffective under the experimental conditions used. Following the three-day treatment interruption, mechanical allodynia was reestablished, although α-PHEL at 100 mg/kg and all doses of α-PHEL/β-CD maintained an antiallodynic effect on the first day after treatment cessation. After treatment was resumed, the antiallodynic effect was again observed until the end of the experimental period.

To explore the mechanisms underlying the antinociceptive effects of α-PHEL, we investigated the potential involvement of opioidergic and serotonergic pathways within the CFA-induced chronic inflammatory pain model. The most effective dose of free α-PHEL (100 mg/kg) was selected for this evaluation, and α-PHEL at this dose produced significant effects from the first hour post-administration.

The administration of naloxone, a non-selective opioid receptor antagonist, significantly reversed the antinociceptive effects of α-PHEL, suggesting that the opioid pathway contributes to its mechanism of action. However, the precise interaction between α-PHEL and opioid receptors remains to be established.

Previous work by Lima et al. [23] also indicated that the antinociceptive action of α-PHEL in acute pain models involved the opioid pathway and K^+^-ATP channels, supporting our findings. The opioid system, crucial for pain modulation, operates through receptors (μ, δ, and κ) distributed in both the central and peripheral nervous systems [50,51].

Interaction with endogenous opioid peptides or exogenous opioid agonists results in reduced nociceptive transmission. Additionally, the nociceptin/orphanin receptor, a recently identified fourth opioid receptor, may also participate in modulating inflammatory pain responses [52]. Our results reinforce the hypothesis that α-PHEL’s antihypernociceptive effect, at least in part, involves modulation of opioid signaling pathways.

The results of this study suggest a possible contribution of the serotonergic system to the antinociceptive effect of α-PHEL. Pretreatment with PCPA, a serotonin synthesis inhibitor, significantly reduced the effect of α-PHEL, providing pharmacological evidence that serotonin synthesis may participate in this response. Similarly, ketanserin significantly reduced the antinociceptive effect of α-PHEL, suggesting the possible involvement of 5-HT_2_A receptors. However, these pharmacological findings do not establish a direct molecular interaction between α-PHEL and serotonergic receptors.

These findings are consistent with experimental evidence demonstrating that descending serotonergic pathways undergo functional changes during persistent CFA-induced inflammatory pain and actively participate in pain modulation [53].

While ketanserin alone did not affect the nociceptive threshold, it significantly reversed the pain-relieving effects of α-PHEL, further indicating the contribution of serotonin to α-PHEL’s efficacy. Because ketanserin preferentially antagonizes 5-HT_2_A receptors, these findings further suggest the participation of this receptor subtype in the antinociceptive action of α-PHEL, although additional studies are required to define its precise contribution [54].

Serotonin is known to play a significant role in the modulation of pain, especially in the descending pain control pathways, which may explain the observed effects. However, its effects can be inhibitory or facilitatory, depending on the receptor subtype involved and the neural circuit recruited [54,55].

This finding aligns with other studies suggesting that serotonin and its receptors participate in central and peripheral pain modulation, supporting the hypothesis of a possible serotonergic contribution to the antinociceptive effect of α-PHEL.

Previous studies have documented the antinociceptive activity of α-PHEL in various experimental pain models [23], including antihyperalgesic effects in a spared nerve injury model of neuropathic pain [56], as well as its anti-inflammatory properties [22]. These anti-inflammatory effects involve the inhibition of neutrophil migration, as well as leukocyte rolling and adhesion, alongside a reduction in the production of the pro-inflammatory cytokines TNF-α and IL-6 [22].

Furthermore, essential oils containing α-PHEL have also demonstrated antinociceptive and anti-inflammatory activities in experimental models [56], and these biological properties have been comprehensively reviewed [57]. Together, these findings provide pharmacological evidence that serotonin synthesis and 5-HT_2_A receptor signaling may contribute to the antinociceptive effect of α-PHEL.

Although the present study demonstrated the antinociceptive potential of α-PHEL and the α-PHEL/β-CD preparation, further studies are needed to better understand the mechanisms involved in the effects observed with α-PHEL/β-CD.

Regarding the safety of the carrier, β-CD was administered orally at 12.5 mg/kg as the vehicle control for the α-PHEL/β-CD preparation. This dose is substantially lower than the oral no-observed-adverse-effect level (NOAEL) of 600 mg/kg body weight/day reported for β-CD in rats [58].

Overall, the physicochemical findings were consistent with an interaction between α-PHEL and β-CD, while the pharmacological experiments showed antinociceptive activity of the α-PHEL/β-CD preparation during repeated administration. The pharmacological evidence suggesting the possible involvement of opioid and serotonergic signaling provides additional information regarding the activity of α-PHEL.

The present study has some limitations. Particle-size measurements and complementary solid-state analyses were not performed; therefore, the available physicochemical findings support, but do not unequivocally confirm, inclusion complex formation. Pharmacokinetic parameters were also not evaluated. In addition, the pharmacological findings were based primarily on behavioral outcomes, without inflammatory biomarker measurements or molecular confirmation of the proposed opioid and serotonergic mechanisms.

## 5. Conclusions

The present study demonstrated that both free α-PHEL and the α-PHEL/β-CD preparation exhibit significant antinociceptive activity in a CFA-induced model of chronic inflammatory pain. α-PHEL effectively reduced mechanical hyperalgesia and mechanical allodynia induced by CFA, with antinociceptive effects observed in both acute and repeated treatment. The α-PHEL/β-CD preparation produced antinociceptive effects at lower administered doses than those used for free α-PHEL during repeated treatment. However, improvements in solubility and oral bioavailability were not directly evaluated and remain to be established.

The pharmacological inhibition experiments suggest that opioid and serotonergic signaling may contribute to the antinociceptive effect of α-PHEL. These findings provide evidence of the possible participation of these pathways but do not establish receptor activation or direct molecular interactions. Further molecular and biochemical studies are required to characterize the mechanisms underlying this effect.

Thus, these results indicate that α-PHEL and its β-CD preparation represent promising candidates for further investigation as antinociceptive agents. However, further studies are necessary to fully elucidate the mechanisms underlying their antinociceptive activity and to assess their clinical applicability.

## Figures and Tables

**Table 1:** Encapsulation efficiency (EE) and loading efficiency (LE) determined for the spray-dried α-phellandrene/β-cyclodextrin (α-PHEL/β-CD) preparation.

	EE (%)	LE (%)
β-Cyclodextrin (α-phellandrene) (10:1)	70.7 ± 1.5%	7.0 ± 0.2%

Data are expressed as mean ± SD (*n* = 3). Abbreviations: EE, encapsulation efficiency; LE, loading efficiency; α-PHEL, α-phellandrene; β-CD, β-cyclodextrin.

**Table 2:** Equivalent α-phellandrene content in the α-PHEL/β-CD preparation doses used in the pharmacological assays.

Dose of α-PHEL/β-CD (mg/kg)	Equivalent α-PHEL Content (mg/kg)
3.12	0.218
6.25	0.438
12.50	0.875

Abbreviations: α-PHEL, α-phellandrene; β-CD, β-cyclodextrin.

**Table 3:** Thermodynamic parameters obtained from molecular modeling of the theoretical α-PHEL/β-CD host–guest system using the PM6 semiempirical method.

Computational Method	Orientations	ΔG (kcal/mol)	K_f_
	P1	2.65	0.01
PM6	P2	−0.17	1.33
	P3	0.47	0.45
	P4	−4.45	1841

Abbreviations: ΔG, Gibbs free energy; K_f_, formation constant; α-PHEL, α-phellandrene; β-CD, β-cyclodextrin.

## Data Availability

The original contributions presented in this study are included in the article. Further inquiries can be directed to the corresponding author.

## Abbreviations

The following abbreviations are used in this manuscript:

α-PHEL	α-Phellandrene
β-CD	β-Cyclodextrin
CFA	Complete Freund’s adjuvant
EE	Encapsulation efficiency
LE	Loading efficiency
PCPA	*p*-Chlorophenylalanine
FTIR	Fourier-transform infrared spectroscopy
SEM	Standard error of the mean
ANOVA	Analysis of variance
IR	Infrared spectroscopy
PM6	Parameterized Method 6
